# Fatigue as a Driver of Overall Quality of Life in Cancer Patients

**DOI:** 10.1371/journal.pone.0130023

**Published:** 2015-06-12

**Authors:** Ryan M. McCabe, James F. Grutsch, Donald P. Braun, Swetha B. Nutakki

**Affiliations:** 1 Medicine & Science, Cancer Treatment Centers of America, Zion, Illinois, United States of America; 2 Department of Epidemiology, University of Illinois School of Public Health, Chicago, Illinois, United States of America; Queen's University Belfast, UNITED KINGDOM

## Abstract

**Background:**

This manuscript describes an approach for analyzing large amounts of disparate clinical data to elucidate the most impactful factor(s) that relate to a meaningful clinical outcome, in this case, the quality of life of cancer patients. The relationships between clinical and quality of life variables were evaluated using the EORTC QLQ-C30 global health domain—a validated surrogate variable for overall cancer patient well-being.

**Methods:**

A cross-sectional study design was used to evaluate the determinants of global health in cancer patients who initiated treatment at two regional medical centers between January 2001 and December 2009. Variables analyzed included 15 EORTC QLQ-C30 scales, age at diagnosis, gender, newly diagnosed/ recurrent disease status, and stage. The decision tree algorithm, perhaps unfamiliar to practicing clinicians, evaluates the relative contribution of individual parameters in classifying a clinically meaningful functional endpoint, such as the global health of a patient.

**Findings:**

Multiple patient characteristics were identified as important contributors. Fatigue, in particular, emerged as the most prevalent indicator of cancer patients’ quality of life in 16/23 clinically relevant subsets. This analysis allowed results to be stated in a clinically-intuitive, rule set format using the language and quantities of the Quality of Life (QoL) tool itself.

**Interpretation:**

By applying the classification algorithms to a large data set, identification of fatigue as a root factor in driving global health and overall QoL was revealed. The ability to practice mining of clinical data sets to uncover critical clinical insights that are immediately applicable to patient care practices is illustrated.

## Introduction

Current Quality of Life (QoL) assessment tools were developed for use in clinical trials to quantify the benefits of innovative therapies on patients’ symptom burden, functioning, and overall quality of life. Clinical investigators have discovered that specific QoL scales provide information on the duration of patient survival independently of known prognostic variables[1,2]. Recent research has determined the clinical significance of changes in QoL scores[3,4]. Additional research has begun to link QoL domains with clinically relevant biological pathways[5–7].

**Fig 1:**
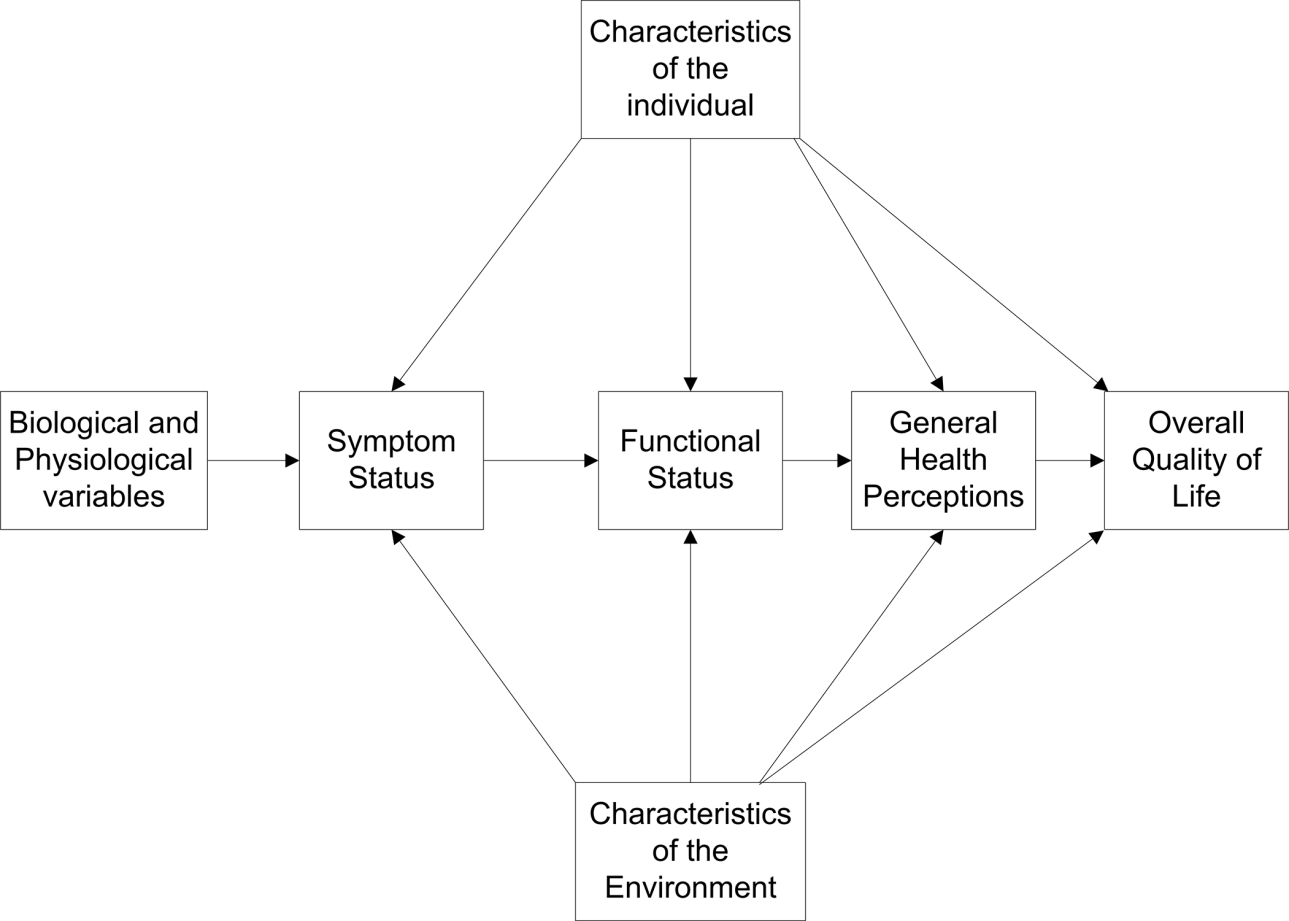
A pathway model of patient quality of life adapted from Wilson & Cleary, 1995. The pathway generally progresses from left to right, starting with the construct of disease state, symptom status, functional status, overall quality of life and patient satisfaction with quality of life. Each construct is composed of multiple patient attributes and is also affected by individual and environmental characteristics.

The emergence of life-extending oncology treatments has resulted in an increasing number of cancer survivors who live for many years after the cessation of treatment. Consequently, the patient’s evaluation of their quality of life and well-being has become an important patient outcome. It is a key driver of patient satisfaction with their clinical team[8] and could increasingly become an important consideration in clinician and patient decision making. The EORTC QLQ-C30 instrument’s modular format replicates the Wilson and Cleary model of health related quality of life (HRQOL) which is a sequence of interconnected constructs that start with physiological and disease state; progress to symptom status, functioning, and general health perception and ends with satisfaction with overall QoL[9–12] (Fig 1).

The main aim of the study is to investigate predictors of QoL using an innovative approach of decision tree analysis. This research used a large database composed of a heterogeneous cancer patient population with Patient Reported Outcomes (PRO), demographic and clinical data. The analysis stratified patients by site of origin, stage of disease and treatment history, i.e., whether they were undergoing first or subsequent lines of therapy. Sets of algorithm-generated decision trees were used to identify the drivers of patient evaluation of their quality of life. Decision trees can generate accurate predictions, handle mixtures of categorical and continuous data, indicate ranges of values where variables are most predictive, and have the advantage that their outputs can be described in clinically intuitive labels, rather than statistical terminology and quantities[13–15]. Decision trees have been successfully used in various scenarios in the medical domain [16,17], including predicting errors in chronic disease care, identifying signals of adverse drug reactions, and detecting artifacts in neonatal ICU data. The use of decision trees to discover drivers of overall quality of life in cancer patients is a novel application. This method enables the investigation of whether the drivers of QoL are diverse and contingent on disease type or are few in number and independent of type of disease and its progression.

## Methods

### Study design

A cross-sectional study design was used to evaluate the determinants of global health in 8478 cancer patients who initiated treatment at two Cancer Treatment Centers of America regional medical centers between January 2001 and December 2009. Administrative staff offered all prospective patients, regardless of treatment or disease history, an opportunity to complete the EORTC QLQ-C30 instrument upon arrival at the clinic, before undergoing treatment. The only criterion for participation was being able to read and complete the survey in English. Demographic data were provided by the cancer registries of each center. All patients gave written consent. This study was approved by the Midwestern Regional Medical Center’s institutional review board.

### QoL Instrument

The EORTC QLQ-C30 is a validated[3] and widely used[18] research instrument that collects Patient-Reported Outcomes (PRO) for symptoms routinely found in cancer patients. It collects data on patient functioning and evaluation of their overall QoL. The instrument consists of 30 questions. The responses to these questions range from 1 to 4 for symptom and functioning domains (1 = Not at all, 4 = Very much) or 1 to 7 for global health domain (1 = Very poor, 7 = Excellent). Responses to all questions are linearly transformed to a 0–100 score in each of 15 categorical (nine symptoms, five functions and one global health), non-overlapping scales (i.e., each response is only used to determine one scale score). The symptoms fatigue, pain, and nausea/vomiting are each composed of multiple questions. For example, fatigue is made up of three questions that ask the patients about their need to rest, feeling weak, and level of tiredness. The remaining symptom scales are single items that address: dyspnea, appetite loss, insomnia, constipation, diarrhea, and the perceived financial effect of the disease and treatment. The five functioning domains are: physical, role (work-related), cognitive, emotional, and social. The global health item combines responses of two questions: patients’ rating of their overall health and overall QoL. For functioning and global health scales, a higher score represents a better level of functioning, while for symptom items, a higher score represents more severe symptoms[19].

### Variables analyzed

The outcome variable of the study was global health, and the goal of the analyses was to identify the structure and accuracy of the trees. This analysis includes all 15 EORTC QLQ-C30 scales, and the following demographic and clinical variables from the cancer registry: age at diagnosis, gender, newly-diagnosed/recurrent disease status, best AJCC (American Joint Committee on Cancer) stage at the time of diagnosis for the analytic patient cohort, and regional/metastatic disease for recurrent cancer patients. These variables were used as inputs to generate classification rules to predict global health levels for individual patients. Gender, newly-diagnosed/recurrent status, and stage were defined as categorical variables.

The clinical variables site of origin, newly-diagnosed or relapsed disease and stage for the newly-diagnosed are powerful predictors of patient lifespan. These variables were used to generate experimental groups with distinct prognoses that ranged from curable to hospice-bound. These subgroups were analyzed independently to determine if the drivers of global health differed by prognosis or whether drivers of global health are independent of site of origin and disease progression.

### Analytic method

The goal of this analysis was to identify determinants of global health from a clinical perspective. Global health scores were stratified into three clinically distinct classes: low, medium, and high. This stratification was based on scores derived from surveys of European general populations[20]. These surveys were population based and conducted without knowledge of the participants’ health status. The stratification of global health score was determined *a priori*. A low global health score corresponded to values that were almost two standard deviations below the general population mean score (~45). A high global health score corresponded to general population mean scores (75) or above[20]. Therefore, patients with scores > 66.67 were defined as high; low ≤ 33.33; medium ranged from 33.33 to and including 66.67[3]. Of the demographic variables used in this analysis, only age at diagnosis was continuous. Decision tree algorithms were parameterized to accommodate the type of each variable (e.g., continuous, categorical, etc.).

### Generating a decision tree

Decision trees (often called Classification and Regression Trees-CART) can be used in multivariate analyses where the underlying distributions of data are unknown or non-normal and the variables are categorical[16]. Decision tree algorithms search the entire dataset to identify the most predictive variable available relative to the target variable (e.g., global health). The algorithm calculates the optimal value of that branching variable to bifurcate the data and maximize classification accuracy. This process is repeated recursively on each split data set until the data are no longer split and a terminal node is generated to classify data in that branch.

This algorithm used Gini coefficients to compute the best splits for each branching node in a given tree[14,21]. The accuracy of a given tree (a goodness-of-fit measure) was computed by coursing a patient-level data record through the branches of the tree until a terminal, leaf node was reached and a classification assigned. The percentage of patients correctly classified was computed for the entire data set.

To ensure the algorithm did not generate a decision tree that was overly specific to a given dataset (i.e., sacrificing generalization), a technique called 10-fold cross-validation was used. Before a tree was generated, a data set was randomly divided into 10 equally sized subsets. The algorithm used the first nine subsets to generate a tree and the held-out set to validate the accuracy of the tree. The cross-validation technique rotated this process through each of the remaining nine subsets for a total of ten iterations. Rather than selecting the most accurate tree from the group, a composite tree was created from the 10 resulting trees[22]. The last step (pruning) reduced the size of the tree by removing branches of the tree that provided little or no improvement in predictive accuracy. Pruning reduces the possibility of over-fitting which might be due to the presence of outliers in the training data. This makes the tree smaller and simpler to generate rule sets. This complete process is run for each clinical subgroup tested.

### How to read a decision tree

**Fig 2:**
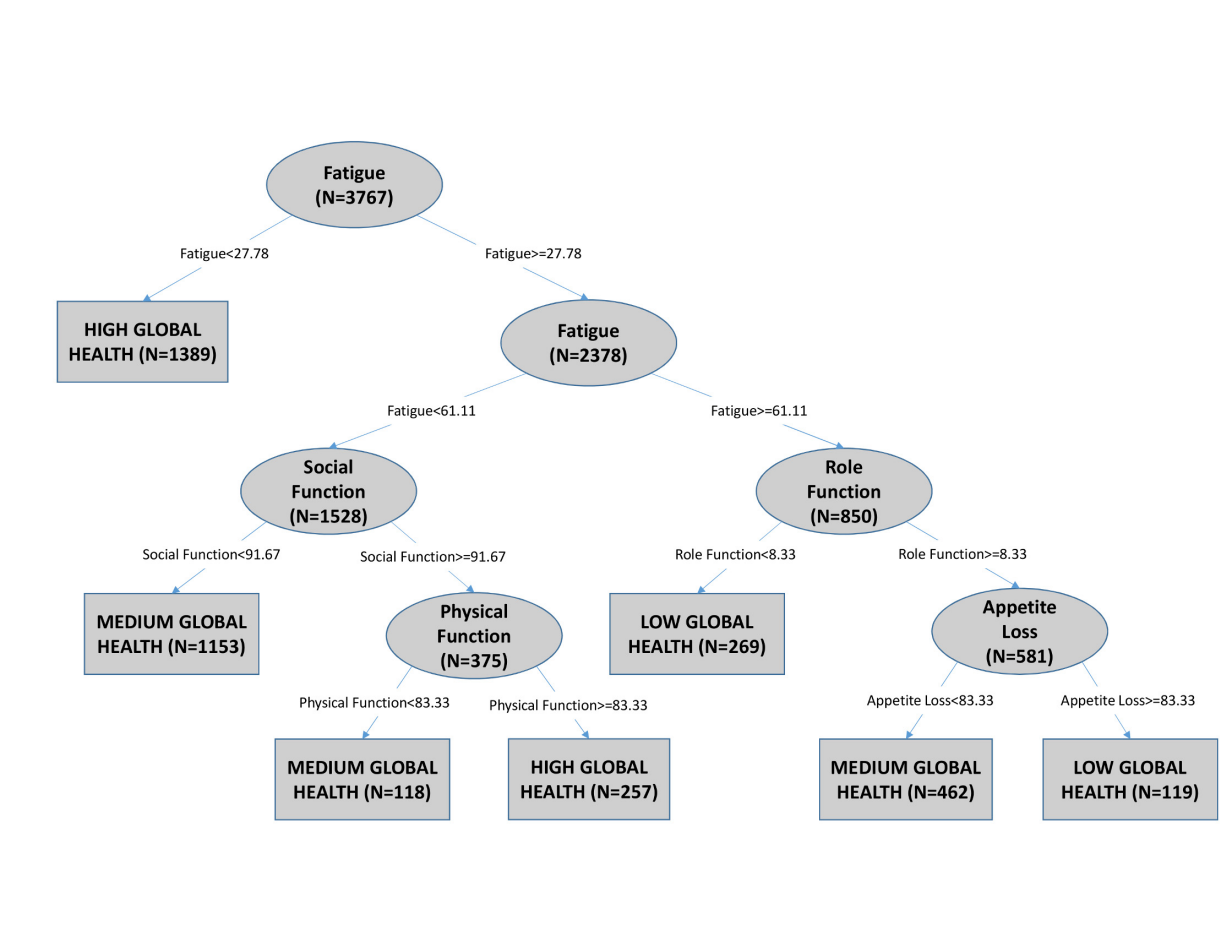
An example decision tree generated from newly diagnosed patients. To predict a patient’s global health level, start at the root node (top oval), traverse the branches–depending on the specific values of Individual patient data–and come to a leaf node (rectangle). The leaf node is the prediction of Low, Medium or High global health for that patient. Paths travelled from the root node to each leaf node can be restated as a conditional rule set listing the drivers of global health levels.

**Fig 3:**
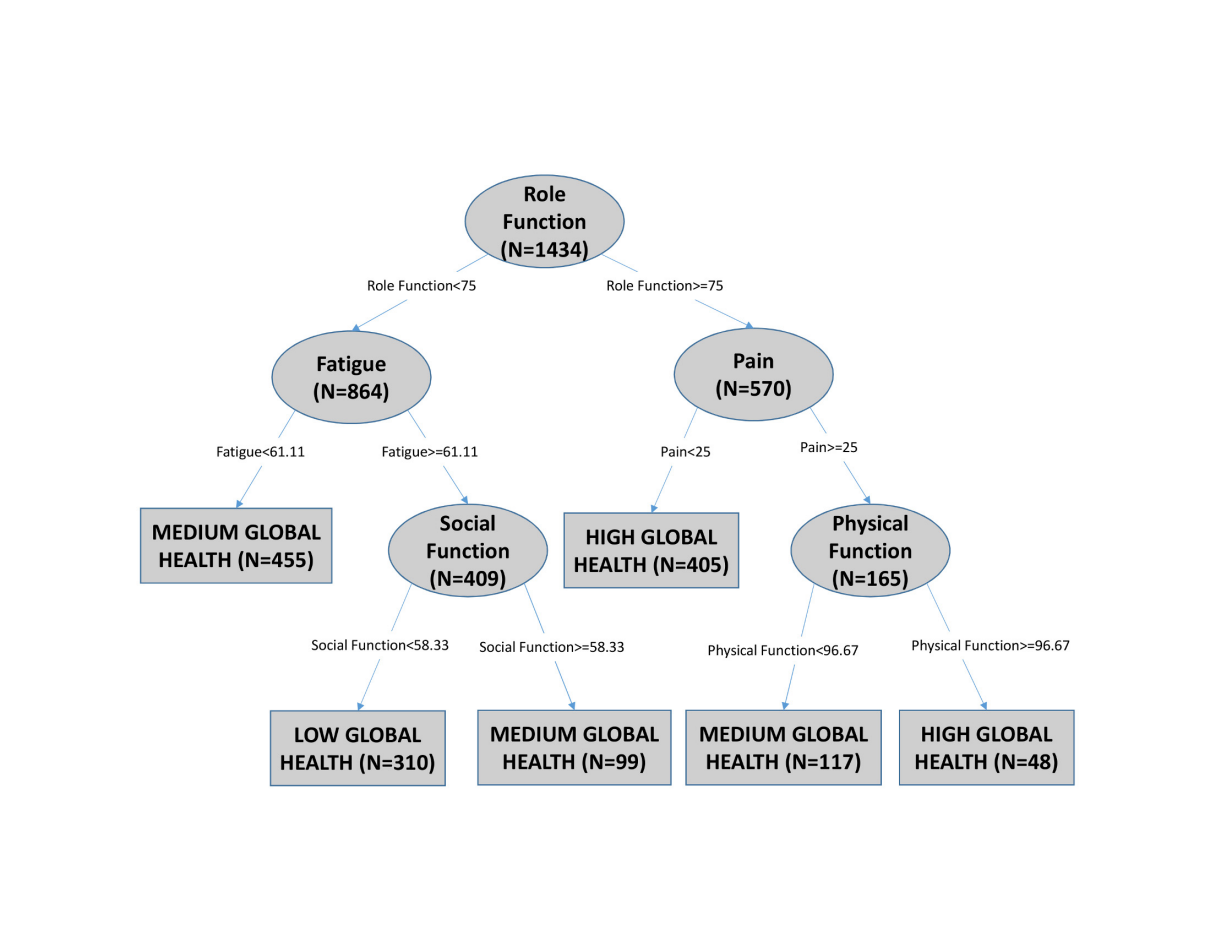
A second example of decision tree generated from newly diagnosed stage 4 patients. This tree has role function as the root node (first split) and fatigue and pain as next splits. ‘N’ in each node represents the number of patients.

Fig 2 is an example of a decision tree generated using the newly-diagnosed patients and Fig 3 is an example generated using newly-diagnosed stage 4 patients from this dataset. In Fig 2, fatigue is the root node, which means that out of all the patient data points under study, fatigue classified global health most accurately if no additional information had been available. The cut point for fatigue at the root node is 27.78. If a given patient had a fatigue score ≥ 27.78, then the right branch would be traversed, and the process repeated with the next node until a classification of global health can be made at a terminal or leaf node. For example, if a patient has a fatigue score < 27.78, then the patient is classified to have high global health with no additional information required. A variable may be selected multiple times as a branching node because different values of that variable, in conjunction with the values of previously selected variables, can contain more information relative to other variables in that subset. In Fig 3, role function is the root node with a cut point of 75.

Classification nodes are terminal nodes that do not split any further. The classification accuracy for a tree is an indication of how much of the structure in the data set the tree has captured. By traversing branches of a decision tree, starting with the root node and ending in a classification node, sets of conditional rules can be identified and restated in clinical terms.

## Results

### Patient Demographics

Cancer registries identified 23,783 potential participants from which 12,357 agreed to complete the instrument preceding initial clinical consultation at two CTCA regional medical centers between January 2001 and December 2009. 11,469 patients returned the questionnaire. The number of respondents who underwent treatment at CTCA and completed the QoL assessment was 8478 patients. The demographics of participants (Table 1) were compared to the non-participants and were found to be similar in prior research[23].

**Table 1:** Patient Characteristics.

Patient Characteristic	Newly Diagnosed	Recurrent Disease
N (8478)	3767 (44.4%)	4711 (55·6%)
Age at study, median	57 ± 10·4	55 ± 10·6
Sex (%)		
Male	1834 (48·7%)	1895 (40·2%)
Female	1933 (51·3%)	2816 (59·8%)
Site of Origin (%)		
Lung	730 (19·4%)	682 (14·5%)
Breast	718 (19·1%)	1102 (23·4%)
Colorectal	243 (6·5%)	628 (13·3%)
Prostate	527 (14·0%)	285 (6·1%)
Pancreatic	415 (11·0%)	292 (6·2%)
All other Cancers	1134 (30·1%)	1722 (36·6%)
Vital Status (%)		
Alive	2009 (53·3%)	1722 (36·6%)
Dead	1758 (46·7%)	2989 (63·4%)
Best AJCC Stage (%)		
Stage 1	470 (12·5%)	69 (1·4%)^a^
Stage 2	908 (24·1%)	186 (3·8%)^a^
Stage 3	644 (17·1%)	258 (5·3%)^a^
Stage 4	1434 (38·1%)	3568 (73·5%)^a^
Unknown Stage	311 (8·3%)	774 (15·9%)^a^

^a^patients re-staged following clinical presentation at CTCA.

This study enrolled patients from all stages of the natural history of diagnosed disease (Table 1). Patients tended to be relatively young for cancer patients, with a majority of participants being female (n = 4505; 56%). Nearly two thirds (65%) of these patients had disease originating in the lung, breast, colon or rectum, prostate, or pancreas. This patient population was biased towards patients who had recurrence of disease. Of the patients presenting with newly-diagnosed disease, over half had stage 3 or 4 disease (55%).

Global health scores were distributed as Low (23%), Medium (43%) and High (34%) classes. Table 2 compares the QOL domain scores of two prognostically distinct cohorts in the study population to a general population cohort[20].

**Table 2:** Baseline Description of Quality of Life (QOL) Scores.

QOL Scale mean ± STD	Newly Diagnosed	Recurrent	EORTC General population
N	3767^a^	4711^a^	7802^b^
Fatigue	38.6 ± 28.1	46 ± 28.6	24.1 ± 24.0
Nausea/vomiting	11.8 ± 20	15.9 ± 23.8	3.7 ± 11.7
Pain	32.1 ± 31	38.4 ± 32.9	20.9 ± 27.6
Dyspnea	21.8 ± 28.4	27.1 ± 30.8	11.8 ± 22.8
Insomnia	37.9 ± 32.3	38.7 ± 32.7	21.8 ± 29.7
Appetite loss	25.6 ± 31.9	29.6 ± 33.7	6.7 ± 18.3
Constipation	20.3 ± 29.2	22.9 ± 30.7	6.7 ± 18.4
Diarrhea	11.2 ± 21.6	13.4 ± 23.5	7.0 ± 18.0
Financial Problems	30.5 ± 33.5	35.1 ± 34.2	9.5 ± 23.3
Physical Function	79.3 ± 22.5	71.3 ± 24.8	89.8 ± 16.2
Role Function	69 ± 32.9	62.8 ± 33.7	84.7 ± 25.4
Emotional Function	65.7 ± 25	66.4 ± 24.8	76.3 ± 22.8
Cognitive Function	78.2 ± 24.2	75.7 ± 25.1	86.1 ± 20.0
Social Function	69 ± 31.8	62.8 ± 32.5	87.5 ± 22.9
Global Health	61.4 ± 25.9	55.4 ± 25.9	71.2 ± 22.4

^a^A total of 8478 patients from 2 CTCA regional medical centers.

^b^Disease free patient population specified in the reference manual of EORTC (Scott et al., 2008).

### Decision Tree

The patient population was stratified into clinical sub-groups by site of origin, newly-diagnosed/relapsed, and AJCC stage for the newly-diagnosed. Each data subset was used to generate a decision tree representing that clinical sub-group. The symptom and functioning scales that were present in each tree as branching nodes are summarized in Table 3. Figs 2 and 3 are examples of decision trees generated from a newly-diagnosed cohort and a newly-diagnosed stage 4 cohort, respectively. The nodes that appear in each tree (row) indicate that the QoL scale (column) was used to classify the level of global health for patients in that clinical sub-group. N represents the number of patients in each node. Any node that appeared in at least one tree was included in Table 3 as a column.

**Table 3:** Summary of decision trees.

Experimental Subgroup		N	Physical	Role	Emotional	Cognitive	Social	Fatigue	Appetite Loss	Pain	Insomnia	Prediction Accuracy (%)	Root Node	Cut point
All Cancers		8478	**×**	**×**			**×**	**×**	**×**			67.34	Fatigue	27.8
Newly Diagnosed		3767	**×**	**×**			**×**	**×**	**×**			68.04	Fatigue	27.8
Recurrent Cancers		4711	**×**	**×**	**×**		**×**	**×**		**×**		66.16	Fatigue	38.9
Newly Diagnosed	Lung	730		**×**	**×**		**×**					67.95	Role	75.0
	Breast	718	**×**	**×**	**×**		**×**	**×**				72.7	Role	91.7
	Colorectal	243					**×**	**×**	**×**			74.49	Fatigue	27.8
	Prostate	527					**×**	**×**				73.62	Fatigue	27.8
	Pancreatic	415			**×**			**×**		**×**		63.61	Fatigue	47.2
	All others	1134	**×**					**×**	**×**			66.84	Fatigue	27.8
Recurrent Cancers	Lung	682			**×**			**×**				67.16	Fatigue	50.0
	Breast	1102	**×**	**×**		**×**	**×**	**×**				68.51	Fatigue	27.8
	Colorectal	628					**×**	**×**				62.26	Fatigue	44.4
	Prostate	285	**×**				**×**	**×**				71.23	Physical	90.0
	Pancreatic	292		**×**	**×**			**×**				69.18	Fatigue	44.4
	All others	1722	**×**	**×**			**×**	**×**	**×**			66.78	Role	75.0
Newly Diagnosed	Stage 1	470	**×**		**×**		**×**	**×**				70.85	Social	91.7
	Stage 2	908		**×**	**×**		**×**	**×**	**×**		**×**	73.68	Role	91.7
	Stage 3	644					**×**	**×**				70.03	Fatigue	27.8
	Stage 4	1434	**×**	**×**			**×**	**×**		**×**		67.5	Role	75.0
	Unknown	311					**×**	**×**				63.99	Fatigue	38.9
Recurrent Cancers	Regional (Stage 1–3)	496		**×**			**×**	**×**				66.13	Fatigue	27.8
	Metastatic (Stage 4)	3470	**×**	**×**	**×**		**×**	**×**		**×**		66.63	Fatigue	38.9
	Unknown	745	**×**	**×**				**×**				66.85	Fatigue	38.9

The two QoL scales that were most commonly found to classify global health were fatigue and social functioning. Fatigue was included in every decision tree except one. The classification accuracies ranged from 62.3–74.5%. The root node for each tree indicated the EORTC QLQ-C30 domain that was selected by the algorithm over the entire data set as containing the most information about global health level; the cut point for the root node was the value of the variable calculated to optimally split the data. Fatigue was the root node in 16 of the 23 trees. Variables not selected by the algorithm for any patient subset were nausea/vomiting, dyspnea, constipation, diarrhea, financial problems, age at diagnosis and stage.

## Discussion

This multivariate analysis was conducted over disparate clinical subgroups to identify QoL domains that had relatively high levels of agreement with overall global health levels. The study cohort was composed of participants whose prognosis ranged from curable to hospice bound and from newly-diagnosed to patients who had already undergone multiple lines of chemotherapy. Nonetheless, all of these patients were still seeking treatment. In this clinically heterogeneous group, the primary driver of global health was fatigue. When patients were further subcategorized by site of origin or tumor stage, fatigue remained the predominant driver of global health across subgroups.

This analysis was facilitated by the use of decision trees. They are easy to understand and interpret and thus have certain advantages over commonly used biostatistics methods. A tree generates a set of conditional rules that can be visualized or written out. Unlike other methods of analysis, decision trees do not depend on the variables to follow any kind of defined, statistical distribution. The variables can be a combination of continuous and categorical values. Decision trees are robust as they are not as affected by outliers. Any outlier would be grouped into a node and thus would have little or no effect on splitting nodes and cut points. Decision trees can work with very large numbers of variables, which is an additional advantage of this methodology[14].

In this analysis global health was categorized as low, medium, or high by anchoring these values to data reported in general population surveys. This categorization was done to frame the analysis in clinically intuitive terms; low score—values that were almost two standard deviations below the general population mean score (~45); high score—general population mean scores (75) or above[20].

Fatigue was identified in every decision tree, representing 23 clinical sub-groups, but one—newly-diagnosed lung. It was the root node for 16/23 patient groups. No other symptom item appeared as the root node. Of the other eight symptom domains in the EORTC QLQ-C30, only pain, appetite loss and insomnia (in one tree) were used to classify global health level. In some trees, the algorithm selected fatigue as a node multiple times. The predominant determinant of global health as identified by the decision tree algorithm was fatigue. These findings extend prior findings that report fatigue as the driver of global health[24,25]. This means that if a clinician could only ask one question of a patient in an attempt to discover their global health level, then they would ask the patient to report their fatigue level.

The value of this finding was underscored by the emergence of fatigue in different patient contexts. The role of fatigue was independent of site of origin, stage of disease, and stage of treatment. By deconstructing the decision trees into rule sets, specific cutpoints where fatigue is a decision node identifies context-specific ranges of fatigue levels. In cases where fatigue was a root node, patients with advanced disease tended to have higher cutpoints (newly-diagnosed-27.8 vs recurrent-38.9), indicating the advanced patient was more fatigued. For the entire study cohort, however, the cutpoint of fatigue was near normal general population levels (27.8 vs 24.1). This suggests that cancer patients with elevated levels of fatigue may improve their overall QoL, if given clinical attention for fatigue.

Fatigue as a clinical symptom remains a complex patient feature. Evidence exists that multiple underlying biological pathways (e.g., depression, insomnia, disruptions of circadian rhythm, and disruption of biological system function), independently or in combination may manifest elevated fatigue levels[7,26,27]. Acknowledging the importance of fatigue as a driver of overall global health levels across cancer patients with different diagnoses, stages of disease and treatment histories is a first step in furthering understanding of these root causes[28,29].

In addition to fatigue, other QOL domains that predicted overall QoL consistently across patient sub-groups were social, role (work-related) and physical functioning. Less common but relevant predictive symptoms for certain patient subgroups included appetite loss, pain, and insomnia. More common constitutional symptoms such as pain and nausea, which are seen frequently in cancer patients might be expected to drive patient perception of quality of life. That this is not the case in the current investigation may reflect a greatly enhanced ability to control such symptoms with the newer generation of pain and anti-emetic medications. Thus, the control of pain and nausea through application of best practice guidelines is expected for the vast majority of patients in our study regardless of treatment history. Unfortunately, symptoms such as fatigue have yet to be adequately controlled in the majority of patients.

Additionally, the emergence of fatigue driving overall QoL followed closely by the importance of functional status domains, combined with the relative lack of observation of commonly reported symptoms as drivers, may in some ways reinforce the Wilson Cleary model that functional status directly drives overall QoL and may also suggest a more complex interpretation that fatigue is something more than an acute, clinical symptom. The items from the EORTC survey that were not selected by the algorithm for any patient subgroups were the symptom items nausea/vomiting, dyspnea, constipation, diarrhea and financial problems. The demographic and clinical variables including age at diagnosis, gender and stage were not identified as predictive of global health by the algorithm for any patient subgroups. It should not be inferred that the patient variables that were not used to classify global health levels in patients were not indicative of patient QoL at all nor that they were not reported as present in patients. Rather, each branching node that was selected by the algorithm to be included in the tree structure was determined to contain the most information over all other variables, given that set patients. Although stage is a powerful predictor of the duration of patient survival, in this patient cohort stage of tumor was not found to be a driver of overall QoL, which is a surprising finding in the world of oncology[2].

This study is limited by the fact that various data points that might be relevant were unavailable at the time of study (specific treatment histories, time from diagnosis, comorbid conditions, performance status and other cancer-specific QoL domains like peripheral neuropathy)[30]. These results confirm the potential value of the inclusion of a question about fatigue in a QoL tool that community oncologists could routinely use in their treatment of patients[31]. The results also emphasize the importance of identifying the biological pathways that directly or indirectly affect the patient’s experience of fatigue; future investigations should include data on biological pathways (e.g., bone marrow suppression, red blood cell count, depression, disruptive circadian rhythms, etc.) that are involved in regulating fatigue[5,6,26,29].

## Conclusion

After stratifying a large patient database into twenty three clinically relevant subsets, fatigue was the most commonly identified domain used to classify global health levels. It is unclear if fatigue is a proxy variable for global health, a direct driver of it, or a driver of functioning domains that, in turn, drive global health. The results of this analysis support the Wilson Cleary theory of QoL but could also support other theoretical models.
